# Supplementary material for: Introduction of regioselective bacterial heme oxygenases into Arabidopsis hy1-1 supports the retrograde heme signaling hypothesis
Source: Plant Physiol. 2026 May 23;201(2):kiag304. doi: 10.1093/plphys/kiag304 (PMC13316938; doi:10.1093/plphys/kiag304)
Supplement: kiag304_Supplementary_Data [file kiag304_supplementary_data.pdf]

***NmHO* coding sequence**

```
        10        20        30        40        50        60
ATGAGAGCTTCTGCTGATTTTGCAAGAACTGCTTCTTTGTCACTTACTGATAAGGGAAAG
M  R  A  S  A  D  F  A  R  T  A  S  L  S  L  T  D  K  G  K

        70        80        90       100       110       120
AACATGTCTGAAACTGAAAACCAAGCTTTGACATTTGCAAAAAGGTTGAAAGCTGATACT
N  M  S  E  T  E  N  Q  A  L  T  F  A  K  R  L  K  A  D  T

       130       140       150       160       170       180
ACTGCTGTTCATGATTCAGTTGATAACCTTGTTATGTCAGTTCAACCTTTCGTTTCTAAG
T  A  V  H  D  S  V  D  N  L  V  M  S  V  Q  P  F  V  S  K

       190       200       210       220       230       240
GAAAATTATATCAAGTTTTTAAAATTGCAGTCTGTTTTCCATAAGGCTGTGGATCACATT
E  N  Y  I  K  F  L  K  L  Q  S  V  F  H  K  A  V  D  H  I

       250       260       270       280       290       300
TATAAGGATGCTGAATTGAACAAGGCTATTCCAGAACTTGAGTATATGGCTAGATATGAT
Y  K  D  A  E  L  N  K  A  I  P  E  L  E  Y  M  A  R  Y  D

       310       320       330       340       350       360
GCTGTTACACAAGATCTTGCTGATCTTGGTGATAAACCTTATGAATACGGAAAACCTTTG
A  V  T  Q  D  L  A  D  L  G  D  K  P  Y  E  Y  G  K  P  L

       370       380       390       400       410       420
CCTCATGAAACAGGAAACAAGGCTATTGGATGGCTTTACTGTGCTGAGGGATCAAACTTG
P  H  E  T  G  N  K  A  I  G  W  L  Y  C  A  E  G  S  N  L

       430       440       450       460       470       480
GGTGCTGCTTTCCTTTTCAAGCACGCTCAAAAGTTGGACTACAATGGTGAGCATGGAGCT
G  A  A  F  L  F  K  H  A  Q  K  L  D  Y  N  G  E  H  G  A

       490       500       510       520       530       540
AGACATCTTGCTCCACATCCTGATGGTAGAGGTAAACATTGGAGAGCTTTCGTTGAACAT
R  H  L  A  P  H  P  D  G  R  G  K  H  W  R  A  F  V  E  H

       550       560       570       580       590       600
CTTAATGCCCTTAACTTGACACCAGAAGCTGAAGCTGAAGCTATCCAAGGTGCCCGTGAG
L  N  A  L  N  L  T  P  E  A  E  A  E  A  I  Q  G  A  R  E

       610       620       630       640       650       660
GCTTTCGCTTTCTATAAGGTTGTGCTTAGAGAAACATTTGGATTGGCAGCTGATGCAGAG
A  F  A  F  Y  K  V  V  L  R  E  T  F  G  L  A  A  D  A  E

       670       680       690
GCACCTGAGGGTATGATGCCTCATAGACATTGA
A  P  E  G  M  M  P  H  R  H  *
```

cNmHO coding sequence

```
        10        20        30        40        50        60
ATGGCTTCCTCTATGCTCTCTTCCGCTACTATGGTTGCCTCTCCGGCTCAGGCCACTATG
M  A  S  S  M  L  S  S  A  T  M  V  A  S  P  A  Q  A  T  M

        70        80        90       100       110       120
GTCGCTCCTTTCAACGGACTTAAGTCCTCCGCTGCCTTCCCAGCCACCCGCAAGATGAGA
V  A  P  F  N  G  L  K  S  S  A  A  F  P  A  T  R  K  M  R

       130       140       150       160       170       180
GCTTCTGCTGATTTTGCAAGAACTGCTTCTTTGTCACTTACTGATAAGGGAAAGAACATG
A  S  A  D  F  A  R  T  A  S  L  S  L  T  D  K  G  K  N  M

       190       200       210       220       230       240
TCTGAAACTGAAAACCAAGCTTTGACATTTGCAAAAAGGTTGAAAGCTGATACTACTGCT
S  E  T  E  N  Q  A  L  T  F  A  K  R  L  K  A  D  T  T  A

       250       260       270       280       290       300
GTTCATGATTCAGTTGATAACCTTGTTATGTCAGTTCAACCTTTCGTTTCTAAGGAAAAT
V  H  D  S  V  D  N  L  V  M  S  V  Q  P  F  V  S  K  E  N

       310       320       330       340       350       360
TATATCAAGTTTTTAAAATTGCAGTCTGTTTTCCATAAGGCTGTGGATCACATTTATAAG
Y  I  K  F  L  K  L  Q  S  V  F  H  K  A  V  D  H  I  Y  K

       370       380       390       400       410       420
GATGCTGAATTGAACAAGGCTATTCCAGAACTTGAGTATATGGCTAGATATGATGCTGTT
D  A  E  L  N  K  A  I  P  E  L  E  Y  M  A  R  Y  D  A  V

       430       440       450       460       470       480
ACACAAGATCTTGCTGATCTTGGTGATAAACCTTATGAATACGGAAAACCTTTGCCTCAT
T  Q  D  L  A  D  L  G  D  K  P  Y  E  Y  G  K  P  L  P  H

       490       500       510       520       530       540
GAAACAGGAAACAAGGCTATTGGATGGCTTTACTGTGCTGAGGGATCAAACTTGGGTGCT
E  T  G  N  K  A  I  G  W  L  Y  C  A  E  G  S  N  L  G  A

       550       560       570       580       590       600
GCTTTCCTTTTCAAGCACGCTCAAAAGTTGGACTACAATGGTGAGCATGGAGCTAGACAT
A  F  L  F  K  H  A  Q  K  L  D  Y  N  G  E  H  G  A  R  H

       610       620       630       640       650       660
CTTGCTCCACATCCTGATGGTAGAGGTAAACATTGGAGAGCTTTCGTTGAACATCTTAAT
L  A  P  H  P  D  G  R  G  K  H  W  R  A  F  V  E  H  L  N

       670       680       690       700       710       720
GCCCTTAACTTGACACCAGAAGCTGAAGCTGAAGCTATCCAAGGTGCCCGTGAGGCTTTC
A  L  N  L  T  P  E  A  E  A  E  A  I  Q  G  A  R  E  A  F

       730       740       750       760       770       780
GCTTTCTATAAGGTTGTGCTTAGAGAAACATTTGGATTGGCAGCTGATGCAGAGGCACCT
A  F  Y  K  V  V  L  R  E  T  F  G  L  A  A  D  A  E  A  P

       790       800
GAGGGTATGATGCCTCATAGACATTGA
E  G  M  M  P  H  R  H  *
```

***PaHO* coding sequence**

```
        10         20         30         40         50         60
ATGGATACACTTGCTCCTGAGTCTACAAGACAAAATCTTAGATCACAGAGACTTAATTTG
M  D  T  L  A  P  E  S  T  R  Q  N  L  R  S  Q  R  L  N  L

        70         80         90        100        110        120
CTTACTAACGAACCTCATCAGCGTTTGGAGTCTCTCGTTAAGTCTAAGGAGCCTTTTGCT
L  T  N  E  P  H  Q  R  L  E  S  L  V  K  S  K  E  P  F  A

       130        140        150        160        170        180
TCAAGAGATAATTTTGCTAGATTCGTTGCTGCTCAATATTTGTTTCAGCATGATTTGGAG
S  R  D  N  F  A  R  F  V  A  A  Q  Y  L  F  Q  H  D  L  E

       190        200        210        220        230        240
CCACTTTATAGAAATGAGGCTCTTGCTAGACTTTTTCCAGATCTTGCTAGTAGAGCTAGA
P  L  Y  R  N  E  A  L  A  R  L  F  P  D  L  A  S  R  A  R

       250        260        270        280        290        300
GATGATGCCGCAAGAGCTGATCTCGCTGATCTTGGTCATGCTGTTCCTGAGGGAGACCAA
D  D  A  A  R  A  D  L  A  D  L  G  H  A  V  P  E  G  D  Q

       310        320        330        340        350        360
TCTGTGAGAGAGGCAGATCTTTCACTTGCTGAGGCTCTCGGTTGGCTTTTCGTTTCTGAG
S  V  R  E  A  D  L  S  L  A  E  A  L  G  W  L  F  V  S  E

       370        380        390        400        410        420
GGTTCTAAATTGGGTGCTGCTTTTTTGTTTAAGAAAGCTGCTGCTCTTGAACTTGATGAA
G  S  K  L  G  A  A  F  L  F  K  K  A  A  A  L  E  L  D  E

       430        440        450        460        470        480
AATTTCGGAGCTAGACATCTTGCTGAGCCTGAGGGAGGAAGAGCTCAAGGATGGAAGTCT
N  F  G  A  R  H  L  A  E  P  E  G  G  R  A  Q  G  W  K  S

       490        500        510        520        530        540
TTTGTTGCAATTTTGGATGGTATCGAGTTGAACGATGAAGAAGAAAGATTGGCAGCTAAA
F  V  A  I  L  D  G  I  E  L  N  D  E  E  E  R  L  A  A  K

       550        560        570        580        590
GGTGCTTCAGATGCATTCAACAGATTTGGTGATCTCCTTGAAAGAACTTTCGCTTGA
G  A  S  D  A  F  N  R  F  G  D  L  L  E  R  T  F  A  *
```

cPaHO coding sequence

```
        10        20        30        40        50        60
ATGGCTTCCTCTATGCTCTCTTCCGCTACTATGGTTGCCTCTCCGGCTCAGGCCACTATG
M  A  S  S  M  L  S  S  A  T  M  V  A  S  P  A  Q  A  T  M

        70        80        90       100       110       120
GTCGCTCCTTTCAACGGACTTAAGTCCTCCGCTGCCTTCCCAGCCACCCGCAAGATGGAT
V  A  P  F  N  G  L  K  S  S  A  A  F  P  A  T  R  K  M  D

       130       140       150       160       170       180
ACACTTGCTCCTGAGTCTACAAGACAAAATCTTAGATCACAGAGACTTAATTTGCTTACT
T  L  A  P  E  S  T  R  Q  N  L  R  S  Q  R  L  N  L  L  T

       190       200       210       220       230       240
AACGAACCTCATCAGCGTTTGGAGTCTCTCGTTAAGTCTAAGGAGCCTTTTGCTTCAAGA
N  E  P  H  Q  R  L  E  S  L  V  K  S  K  E  P  F  A  S  R

       250       260       270       280       290       300
GATAATTTTGCTAGATTCGTTGCTGCTCAATATTTGTTTCAGCATGATTTGGAGCCACTT
D  N  F  A  R  F  V  A  A  Q  Y  L  F  Q  H  D  L  E  P  L

       310       320       330       340       350       360
TATAGAAATGAGGCTCTTGCTAGACTTTTTCCAGATCTTGCTAGTAGAGCTAGAGATGAT
Y  R  N  E  A  L  A  R  L  F  P  D  L  A  S  R  A  R  D  D

       370       380       390       400       410       420
GCCGCAAGAGCTGATCTCGCTGATCTTGGTCATGCTGTTCCTGAGGGAGACCAATCTGTG
A  A  R  A  D  L  A  D  L  G  H  A  V  P  E  G  D  Q  S  V

       430       440       450       460       470       480
AGAGAGGCAGATCTTTCACTTGCTGAGGCTCTCGGTTGGCTTTTCGTTTCTGAGGGTTCT
R  E  A  D  L  S  L  A  E  A  L  G  W  L  F  V  S  E  G  S

       490       500       510       520       530       540
AAATTGGGTGCTGCTTTTTTGTTTAAGAAAGCTGCTGCTCTTGAACTTGATGAAAATTTC
K  L  G  A  A  F  L  F  K  K  A  A  A  L  E  L  D  E  N  F

       550       560       570       580       590       600
GGAGCTAGACATCTTGCTGAGCCTGAGGGAGGAAGAGCTCAAGGATGGAAGTCTTTTGTT
G  A  R  H  L  A  E  P  E  G  G  R  A  Q  G  W  K  S  F  V

       610       620       630       640       650       660
GCAATTTTGGATGGTATCGAGTTGAACGATGAAGAAGAAAGATTGGCAGCTAAAGGTGCT
A  I  L  D  G  I  E  L  N  D  E  E  E  R  L  A  A  K  G  A

       670       680       690       700       710
TCAGATGCATTCAACAGATTTGGTGATCTCCTTGAAAGAACTTTCGCTTGA
S  D  A  F  N  R  F  G  D  L  L  E  R  T  F  A  *
```

Supplementary Figure S1

Optimized nucleotide sequences of *NmHO*, *cNmHO*, *PaHO*, and *cPaHO*. Based on the original amino acid sequences (Ratliff et al., 2001, Supplementary Table 2), codon-optimized nucleotide sequences for *Arabidopsis* were generated by VectorBuilder (https://en.vectorbuilder.com/). In the cases of *cNmHO* and *cPaHO*, the transit peptide sequence of the *Arabidopsis RBCS* (green) was inserted before the initiation codon.

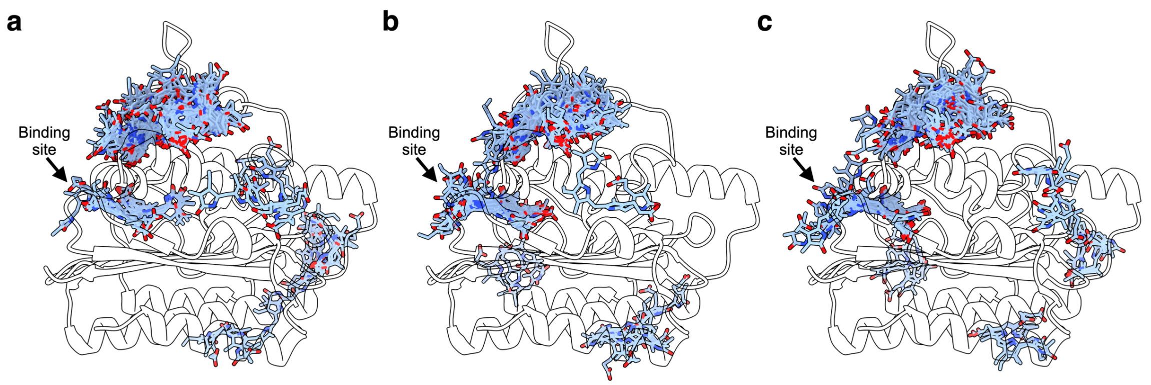

Supplementary Figure S2

Docking simulation of HY2 with biliverdin isomers under the unrestricted condition. Docking models of Biliverdin IXα (a), IXβ (b), and IXδ (c) obtained without spatial restriction (unrestricted docking) in HY2.

Supplementary Table S1: List of primers.

Name	Sequence	Description
Pa-F1	CACCATGGATACACTTGCTCCTGAG	PCR amplification with Pa-F1, Pa-R1 and Nm-F1, Nm-R1 with pEF constructs as templates for cloning with pENTR dTOPO cloning kit
Pa-R1	AGCGAAAGTTCTTTCAAGGAGATC	
Nm-F1	CACCATGAGAGCTTCTGCTGATTTTGC	
Nm-R1	ATGTCTATGAGGCATCATACCCTC	
Pa Chlt-F1	AGCAAGTGTATCCATCTTGCGGGTGGCTGGGAAG	PCR amplification with Pa Chlt F1-R1 and Nm Chlt F1-R1 with pENTR-Chlt yHS as template.
Pa Chlt-R1	GAAAGAACTTTCGCTAAGGGTGGGCGCGCCGACCCAGC	
Nm Chlt-F1	AGCAGAAGCTCTCATCTTGCGGGTGGCTGGGAAG	
Nm Chlt-R1	ATGCCTCATAGACATAAGGGTGGGCGCGCCGACCCAGC	
Pa-F2	CCAGCCACCCGCAAGATGGATACACTTGCTCCTGAG	PCR amplification with Pa_PigA and Nm_HO with Pa-F2, R2 and Nm-F2, R2. then that fragments carried out infusion reaction with each fragment that PCR amplification with Pa Chlt F1-R1 and Nm Chlt F1-R1 with pENTR-Chlt yHS as template.
Pa-R2	GGCGCGCCCACCCTTAGCGAAAGTTCTTTCAAGGAGATC	
Nm-F2	CCAGCCACCCGCAAGATGAGAGCTTCTGCTGATTTTGC	
Nm-R2	GGCGCGCCCACCCTTATGTCTATGAGGCATCATACCCTC	
HY2-F	ATGGGTCGCGGATCCGTCTCTGCTGTGTCGTAT	PCR amplification of HY2-F and HY2-R with RIKEN full-length cDNA clone as a template. PCR amplification of pET28a-F and pET28a-R with pET28a vector as template. Both fragments are ligated by Gibson cloning.
HY2-R	TGGTGGTGGTGGTGCTCGAGTTATTAGCCGATAAATTGTCCT	
pET28a-F	CTCGAGCACCACCACCACCA	
pET28a-R	GGATCCGCGACCCATTTGCTG	
chlt_F	ATGGCTTCCTCTATGCTCTCTTC	for checking infections to Arabidopsis thaliana hy1-1
NmHO_qpF	GCACGCTCAAAAGTTGGACT	for qPCR
NmHO_qpR	TCTGGTGTCAAGTTAAGGGCA	for qPCR

PigA_qpF	CGAACCTCATCAGCGTTTGG	for qPCR
PigA_qpR	TGCGGCATCATCTCTAGCTC	for qPCR
Chlt_qpF	ATGGCTTCCTCTATGCTCTCT	for qPCR
Chlt_qpR	GGAAGGCAGCGGAGGACTTA	for qPCR
ACT8_F	ACTGTGCCTATCTACGAGGGTTTC	for qPCR (Chen et al., 2024))
ACT8_R	CCCGTTCTGCTGTTGTGGT	for qPCR (Chen et al., 2024))
LHCA4-F	AACCCGCTTAACTTTGCTCCTAC	for qPCR (Chen et al., 2024))
LHCA4-R	CAAACCCTAAGAATGCCAACATC	for qPCR (Chen et al., 2024))
LHCB1.1-F	GAGCCAAGTTCTATCTGTTTG	for qPCR (Chen et al., 2024))
LHCB1.1-R	TCTACCATCCACCACAAACAC	for qPCR (Chen et al., 2024))
LHCB1.2-F	GATGGGAGCTGTTGAAGGCT	for qPCR (Chen et al., 2024))
LHCB1.2-R	CCTCTGGGTCGGTAGCAAGA	for qPCR (Chen et al., 2024))
PSBQA-F	AATGGCTCTGGAAGAGTGGC	for qPCR (Chen et al., 2024))
PSBQA-R	AATAGCATCGGCGAGGACAG	for qPCR (Chen et al., 2024))
FC1_F	TGCAGTGGTTGACTCAGAAGATAGCGAG	for qPCR
FC1_R	TTTGGGGAGAGAAGTAGGACGAAAGCCAG	for qPCR

Supplementary Table S2: Accession numbers for amino acid sequences.

Gene	Genbank/EMBL accession number
Neisseria meningitidis HemO	WP_238024207.1
Pseudomonas aeruginosa PigA	NPZ76626.1
